# High-performance activated carbons from *Canarium schweinfurthii* and *Ricinodendron heudelotii* shells for the efficient removal of indigo carmine from water

**DOI:** 10.1039/d6ra00571c

**Published:** 2026-04-21

**Authors:** Daouda Kouotou, Frank Dorinel Feudjio Solefack, Abdelhakim Elmouwahidi, Murat Yilmaz, Julius Nsami Ndi, Agustin F. Perez-Cadenas, Francisco Carrasco-Marin

**Affiliations:** a Applied Physical and Analytical Chemistry Laboratory, Department of Inorganic Chemistry, Faculty of Science, University of Yaoundé I PO. Box 812 Yaoundé Cameroon kouotoudaouda@gmail.com; b Materiales Polifuncionales Basados en Carbono (UGR-Carbon), Departamento de Química Inorgánica, Unidad de Excelencia Química Aplicada a Biomedicina y Medioambiente, Universidad de Granada (UEQ-UGR) ES18071 Granada Spain; c Department of Chemistry and Chemical Processing Technologies, Osmaniye Korkut Ata University PO Box 80010 Osmaniye Turkey

## Abstract

In this study, two biomass wastes, namely, *Canarium schweinfurthii* shells (CSS) and *Ricinodendron heudelotii* shells (RHS), were converted into activated carbons by chemical activation using phosphoric acid. The two prepared adsorbents were designated as AC-CSS-H_3_P and AC-RHS-H_3_P, representing the activated carbons derived from CSS and RHS, respectively, through H_3_PO_4_ activation. The samples were characterized using N_2_ adsorption (BET), scanning electron microscopy (SEM), and Fourier-transform infrared (FTIR) spectroscopy analyses. To achieve the removal of indigo carmine (IC) in the batch mode, four adsorption parameters, namely, contact time (5–30 min), pH (2–11), adsorbent dose (10–110 mg), and initial dye concentration (5–25 mg L^−1^), were thoroughly investigated. Four adsorption isotherms and kinetic models were also evaluated. The specific surface area and pore volume were determined to be 1612 m^2^ g^−1^ and 0.78 cm^3^ g^−1^ for AC-CSS-H_3_P and 1696 m^2^ g^−1^ and 0.90 cm^3^ g^−1^ for AC-RHS-H_3_P, respectively, with a predominance of acidic functional groups for both samples. After 10 minutes of the contact time with 10 mg of the adsorbent in 100 mL of the IC solution (10 mg L^−1^), the IC removal efficiency reached approximately 95%. The adsorption of IC was best described by the Langmuir isotherm model, with maximum adsorption capacities of 588 mg g^−1^ and 1250 mg g^−1^ for AC-CSS-H_3_P and AC-RHS-H_3_P, respectively. The pseudo-second-order kinetic model provided the best fit, with correlation coefficients (*R*^2^) close to unity, indicating a chemisorption mechanism. The adsorption process was predominantly facilitated by hydrogen bonding between the acidic functional groups on the adsorbent surfaces and the anionic form of IC under acidic conditions. Conversely, IC removal decreased in alkaline media due to strong electrostatic repulsion between hydroxide ions and the IC anion. After six regeneration cycles, both adsorbents retained most of their adsorption capacity, demonstrating good reusability. Overall, these results indicate that the activated carbons derived from CSS and RHS are effective adsorbents for the elimination of organic dyes from aqueous solutions.

## Abstract 2

In this study, two biomass wastes, namely, *Canarium schweinfurthii* shells (CSS) and *Ricinodendron heudelotii* shells (RHS), were converted into activated carbons by chemical activation using phosphoric acid.

## Graphical abstract

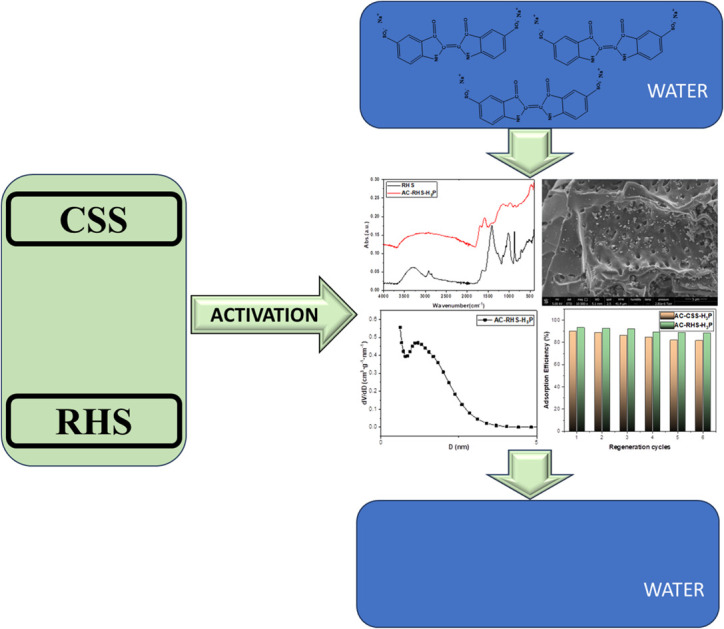

## 1. Introduction

Over the past two decades, dyeing activities have intensified in both artisanal and industrial environments.^1–6^ This increase is largely attributed to the wide availability and use of various natural and synthetic dyes across numerous sectors, including textiles, leather, plastics, printing, food, cosmetics, and pharmaceuticals.^7–12^ While this industry provides an important source of income for many individuals, it has simultaneously become a global concern due to the significant environmental consequences associated with dye effluents.^13–17^ There is no doubt about the extensive use of dyes in daily life; however, the major concern lies in the large quantities of untreated dye-containing effluents discharged into the environment.^1,7,8,10^ The direct release of dye effluents without prior treatment is considered a poor environmental practice. It poses serious risks to human health and, more critically, to aquatic ecosystems, as dye pollutants reduce the light penetration into water bodies, significantly limiting photosynthesis and consequently causing substantial ecological damage.^11–16,18,19^ Synthetic dyes, even at low concentrations, are challenging to biodegrade because of their intricate aromatic structures, which provide significant physicochemical, thermal, and optical stability. These characteristics allow the dyes to persist in wastewater, posing a continuous risk to human and animal health if not properly removed.^10–14,20^ Moreover, many dyes have been reported to exhibit mutagenic and carcinogenic properties, along with other adverse health effects.^1,8,10–12^ Among these hazardous dyes, IC (acid blue 74) is widely used in the textile industry and related sectors.^4–6,15–17^ The selection of IC for this study is particularly relevant, as the global demand for dyed denim fabrics continues to rise, especially in African countries. Consequently, large quantities of IC-containing effluents are discharged into the environment by textile industries, creating increasing environmental and public-health concerns.^4,10–14,16,17,21,22^ The widespread use of the IC dye in the textile industry is largely attributed to its chemical properties. IC is an anionic dye containing two sulfonic groups, which enhance its solubility in water and other solvents by promoting interactions with hydroxyl-containing fibers.^10–15^ If the IC dye is not effectively removed from domestic or industrial effluents, it can persist in aquatic systems, continuously contaminating surface waters and potentially infiltrating groundwater.^5,11,13,23–25^ Therefore, given their recognized harmful effects on the environment, it is imperative to treat IC-contaminated effluents prior to discharge.

To safeguard water resources, numerous efforts have been undertaken to address IC dye pollution, as documented in scientific reports.^10–13,21,23–25^ These initiatives aim to significantly reduce dye concentrations to levels deemed acceptable by the WHO and other environmental organizations. Similarly, various adsorbents have been employed to mitigate IC dye pollution, including carbon-based materials,^5,6,10–13,22,24,26^ clay materials,^15^ and activated carbon.^12,14,21,23,27–33^ Currently, several methods have been implemented to remove colored compounds, including chemical and physicochemical approaches such as coagulation and flocculation,^34^ reverse osmosis,^35^ photochemical degradation,^36–39^ membrane filtration,^40,41^ and adsorption.^4,10–12,14,16,17,21–23,29,30,42^ Among the adsorbents and remediation methods, adsorption by activated carbons (ACs) is considered the most efficient, eco-friendly, and economic. Their main advantages include flexible design, simple preparation, and convenient operation, in contrast to other methods, which are often costly and require large amounts of chemical reagents.^10–12,14,21,43–46^ There is also growing interest in using renewable biomass residues for the preparation of activated carbons. Biomass residues are abundant, readily available, and serve as excellent precursors for producing ACs with desirable physicochemical properties, which are particularly effective for dye removal.^12,21,23,27,28,47–49^

Building on the aforementioned considerations, the present study focuses on the use of two biomass wastes, *Canarium schweinfurthii* shells (CSS) and *Ricinodendron heudelotii* shells (RHS), which have been scarcely explored as precursors, for activated carbon production. These biomass residues are abundant in subtropical countries such as Cameroon, where they are generated in large quantities. Moreover, CSS and RHS possess favorable characteristics for adsorbent preparation, including high availability and low ash content, making them suitable precursors for AC synthesis.^23,50,51^ Furthermore, the chemical activation of lignocellulosic precursors, particularly with phosphoric acid, enhances their mesoporosity, which is advantageous for the adsorption of large dye molecules.^52,53^ Given the chemical structure of IC dye, it qualifies as a large molecule and is therefore suitable for removal using such mesoporous activated carbons. The originality of this work lies in two aspects. First, it utilizes two different lignocellulosic precursors that undergo an acid pretreatment prior to chemical activation under mild-temperature conditions. Second, the resulting activated carbons are employed for the removal of IC dye, an application that has been scarcely explored using these types of biomass waste. Therefore, the overall objective of this study is to add value to these two precursors by converting them into activated carbons and evaluating their adsorption performance for IC dye in aqueous solutions.

## 2. Materials and methods

### 2.1. Chemicals

**Fig. 1:**
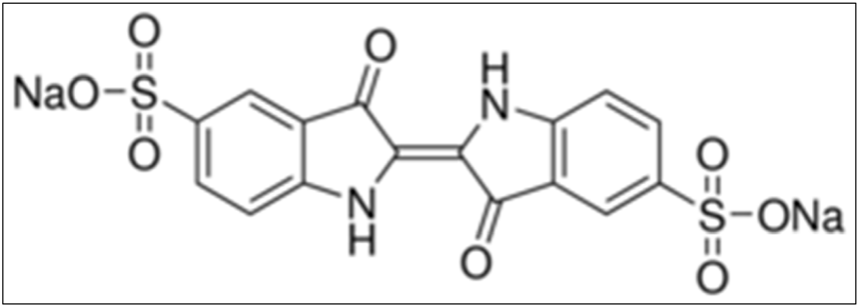
Chemical structure of IC.

IC dye, or acid blue 74, has a chemical formula of C_16_H_8_N_2_Na_2_O_8_S_2_, a color index of 73 015, and a molecular weight of 466.35 g mol^−1^. It was purchased from Sigma-Aldrich Chemie GmbH. Its chemical structure is shown in Fig. 1. Other chemicals, including sulfuric acid (95%), sodium hydroxide (85.5%), hydrochloric acid (37%), and phosphoric acid (85%), were obtained from AnalaR NORMAPUR. All compounds were of analytical quality and utilized without additional purification.

### 2.2. Activated carbon preparation

Exactly 10 g of each precursor (CSS and RHS, sieved to 800 µm) was mixed with 7 M H_2_SO_4_ and subjected to reflux heating at 80 °C for 8 hours. Each pretreated sample was then washed and oven-dried at 110 °C for 24 hours. The pretreated samples were subsequently impregnated with H_3_PO_4_ at a mass impregnation ratio of 3 : 2 and oven-dried at 110 °C for an additional 24 hours. Following impregnation, a specific quantity of each sample was placed in the reactor of a tubular furnace (CARBOLITE 1200 °C tube furnace, KEISON Products) and heated at a rate of 5 °C min^−1^ to a final temperature of 450 °C, maintained for 2 hours under a nitrogen flow at 100 mL min^−1^, and then allowed to cool completely in the furnace. The pyrolyzed samples were initially rinsed with hot water and then with distilled water until the effluents attained neutral pH. The washed samples were then oven-dried at 110 °C for 24 hours. After drying, the activated carbon samples were removed from the oven, crushed into powders, and stored in glass bottles for further use. The samples were designated as AC-CSS-H_3_P and AC-RHS-H_3_P, representing activated carbons derived from CSS and RHS *via* H_3_PO_4_ activation, respectively.

### 2.3. Characterization of activated carbons

Surface and volume characteristics were determined by N_2_ adsorption–desorption using a Micromeritics ASAP 2020 analyzer. The specific surface area was determined using the BET method, the pore volume using the Dubinin–Radushkevich method, and the pore size distribution using the QSDFT method. The surface morphology was investigated using scanning electron microscopy (SEM, JEOL JSM-IT200), and the functional groups on the activated carbon surfaces were subjectively assessed *via* Fourier transform infrared (FTIR) spectroscopy (Vertex 70 DTGS).

### 2.4. Adsorption experiments

Batch adsorption experiments of IC onto AC-CSS-H_3_P and AC-RHS-H_3_P were performed by altering the contact time, pH, adsorbent mass, and initial dye concentration to investigate adsorption isotherms and kinetics. The IC stock solution was prepared by dissolving 100 mg of the IC powder in 1000 mL of distilled water, and working solutions were subsequently prepared by dilution. All adsorption experiments were performed at room temperature (23 °C). The IC concentration in the solution was determined using a calibration curve obtained from absorbance measurements of standard IC solutions at *λ*_max_ = 614 nm using a UV-vis spectrophotometer (DR 5500). The amount of IC adsorbed was calculated from the solution concentrations before and after adsorption, expressed as the quantity adsorbed at equilibrium (*Q*_e_), at time *t* (*Q*_*t*_), and as the percentage removal (% removal), using the respective equations given below:1
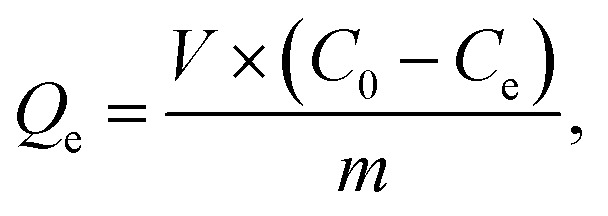
2
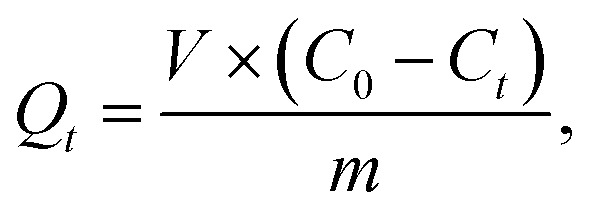
3
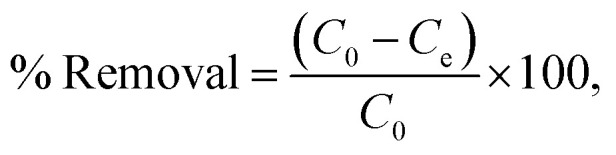
where *C*_0_, *C*_e_, and *C*_*t*_ (mg L^−1^) represent the initial, equilibrium, and time-dependent concentrations of IC, respectively; *V* (l) denotes the volume of the IC solution; and *m* (g) signifies the mass of the adsorbent (AC-CSS-H_3_P or AC-RHS-H_3_P).

### 2.5. Adsorption isotherm models

The experimental results were analyzed using four established isotherm models: Langmuir, Freundlich, Temkin, and Dubinin–Radushkevich (D–R). The equations for these isotherms, in their linear forms, are presented in Table 1.

**Table 1:** Adsorption isotherms and their linear transformations

Isotherm models	Linear equations	Parameters	References
Langmuir	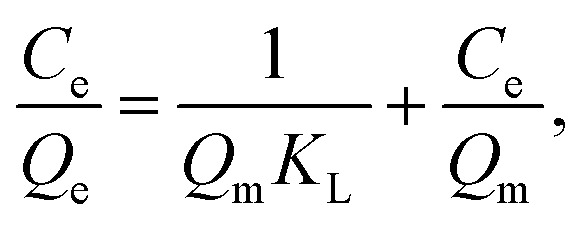	*Q*_m_ and *K*_L_	54
Freundlich	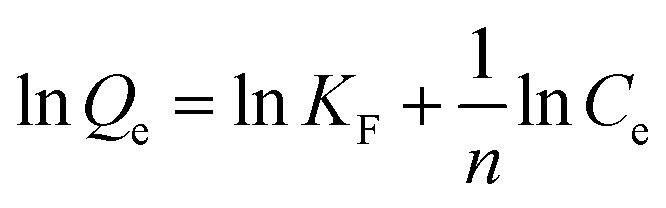	*K*_F_ and *n*	55 and 56
Temkin	*Q*_e_ = *B*_T_ ln *C*_e_ + *B*_T_ ln *K*_T_	*B* and *K*_T_	57
D–R	ln *Q*_e_ = −*B*_D_*ε*^2^ + ln *Q*_D_	*B*_D_ and *Q*_D_	58 and 59

Here, *C*_e_ (mg L^−1^) denotes the equilibrium concentration, *Q*_m_ (mg g^−1^) represents the maximum monolayer adsorption capacity, and *K*_L_ (L mg^−1^) signifies the Langmuir constant related to the adsorption energy. The parameters *Q*_m_ and *K*_L_ are obtained from the plot of 1/*Q*_e_ versus 1/*C*_e_. In addition, the dimensionless separation factor, *R*_L_, serves to assess the viability of adsorption^60^ and is calculated using the following equation:4
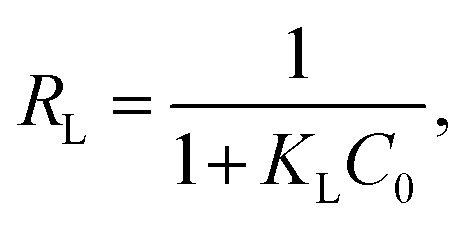
where *C*_0_ (mg L^−1^) represents the initial solute concentration. For the Langmuir separation factor, *R*_L_, *R*_L_ = 0 indicates irreversible adsorption, 0 < *R*_L_ < 1 indicates favorable adsorption, *R*_L_ = 1 corresponds to linear adsorption, and *R*_L_ > 1 indicates unfavorable adsorption. For the Freundlich isotherm, *K*_F_ (mg g^−1^) denotes the Freundlich constant, representing the adsorption capacity, and 1/*n* indicates the adsorption intensity. The graph of ln *Q*_e_*versus* ln *C*_e_ is utilized to ascertain the Freundlich constants *K*_F_ and *n*. For the Temkin isotherm model, *B* (J mol^−1^) represents the heat of adsorption, *K*_T_ (L g^−1^) is the Temkin equilibrium binding constant, *R* (8.314 J mol^−1^ K^−1^) is the universal gas constant, and *T* (K) indicates the absolute temperature. The parameters *B* and *K*_T_ are obtained from the plot of *Q*_e_*versus* ln *C*_e_. For the Dubinin–Radushkevich (D–R) isotherm model, *Q*_D_ (mg g^−1^) is the D–R constant and *B*_D_ (mol^2^ kJ^−2^) is related to the mean free energy of adsorption. These parameters were determined from the plot of ln *Q*_e_*versus ε*^2^, where *ε* is the Polanyi potential, given by the following equation:5
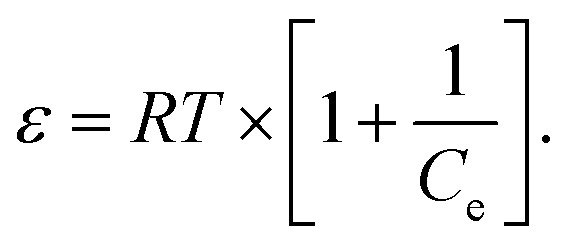


### 2.6. Adsorption kinetics models

Adsorption kinetics provide insights into the controlling reaction pathways and the mechanisms involved in the adsorption process. It also allows for the determination and prediction of the rate at which the target pollutant is removed from the medium. In this study, the experimental data were fitted to four classic kinetic models: pseudo-first order (PFO), pseudo-second order (PSO), Elovich, and intraparticle diffusion. The linear representations of these models are summarized in Table 2.

**Table 2:** Adsorption kinetics and their linear transformations

Kinetic models	Linear equations	Parameters	References
PFO	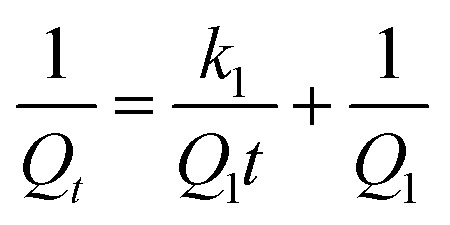	*k*_1_ and *Q*_1_	61 and 62
PSO	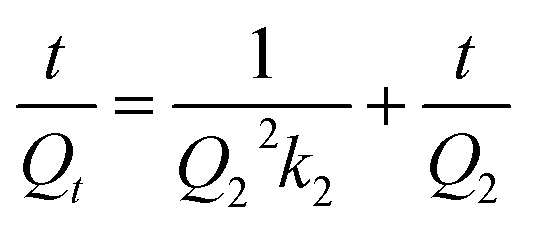	*k*_2_ and *Q*_2_	63 and 64
Elovich	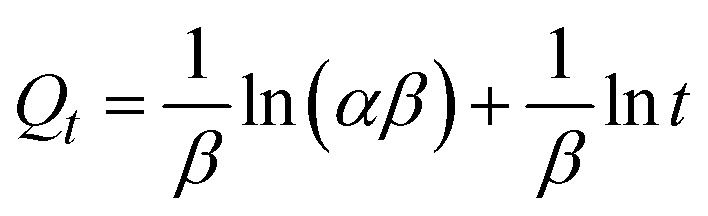	*α* and *β*	65
Intraparticle diffusion	*Q*_*t*_ = *k*_P_*t*^1/2^ + *C*	*k*_P_ and *C*	66

In the pseudo-first-order (PFO) model, *Q*_1_ (mg g^−1^) and *Q*_*t*_ (mg g^−1^) represent the adsorption capacity at equilibrium and the amount adsorbed at time *t*, respectively, while *k*_1_ (min^−1^) is the PFO rate constant. The parameters *Q*_1_ and *k*_1_ are determined from the plot of ln(*Q*_e_ − *Q*_*t*_) *versus t*. In the pseudo-second-order (PSO) model, *Q*_2_ (mg g^−1^) is the adsorption capacity at equilibrium, and *k*_2_ (g mg^−1^ min^−1^) is the PSO rate constant, obtained from the plot of *t*/*Q*_*t*_*versus t*. In the Elovich model, *α* (mg g^−1^ min^−1^) is the initial adsorption rate, and *β* (g mg^−1^) is the desorption coefficient; these parameters are determined from the plot of *Q*_*t*_*versus* ln *t*. In the intraparticle diffusion model, *k*_P_ (mg (g min^1/2^)^−1^) is the intraparticle diffusion rate constant, and *C* (mg g^−1^) is the intercept reflecting the boundary layer effect; both are obtained from the plot of *Q*_*t*_*versus t*^1/2^.

## 3. Results and discussion

### 3.1. Characterization results of the precursors, AC-CSS-H_3_P and AC-RHS-H_3_P

#### 3.1.1. Proximate and elemental analyses

The results of the proximate and elemental analyses of the prepared activated carbons are summarized in Table 3. According to the proximate analysis, CSS contains 5.23% moisture, 68.07% volatile matter, 4.31% ash, and 22.32% fixed carbon, whereas RHS contains 5.07% moisture, 63.53% volatile matter, 3.25% ash, and 28.15% fixed carbon. According to the elemental analysis, CSS is primarily composed of carbon (61.86%), hydrogen (4.36%), nitrogen (3.05%), and oxygen (30.76%), while RHS contains carbon (60.78%), hydrogen (3.46%), nitrogen (2.95%), and oxygen (33.86%). The ash contents, nitrogen levels, and carbon levels in CSS and RHS are comparatively higher than those reported for coconut shells and shea nut shells, while the other components are similar. The CSS and RHS precursors exhibit low ash contents and high carbon contents, indicating that they are suitable raw materials for activated carbon production.

**Table 3:** Proximate and elemental analyses of AC-CSS-H_3_P and AC-RHS-H_3_P

Property	Percentage (wt%)				ASTM test standard
	CSS (this study)	RHS (this study)	Sherry stone shells^67^	Coconut shells^68^	
**Proximate analysis**					
Moisture	5.23	5.07	3.90	5.62	D 1762-84
Volatiles	68.07	63.53	82.78	74.9	D 5832-98
Ash	4.31	3.25	0.40	0.70	D 2866-11
Fixed carbon^a^	22.32	28.15	16.82	24.40	

**Elemental analysis**					
Carbon	61.86	60.78	52.76	53.90	
Hydrogen	4.36	3.46	6.18	5.70	
Nitrogen	3.05	2.95	0.76	0.10	
Sulphur	n.d^b^	n.d^b^	0.02	0.02	
Oxygen^a^	30.76	33.86	40.28	39.44	

a By difference.

b No detection.

#### 3.1.2. FT-IR spectra of AC-CSS-H_3_P and AC-RHS-H_3_P

**Fig. 2:**
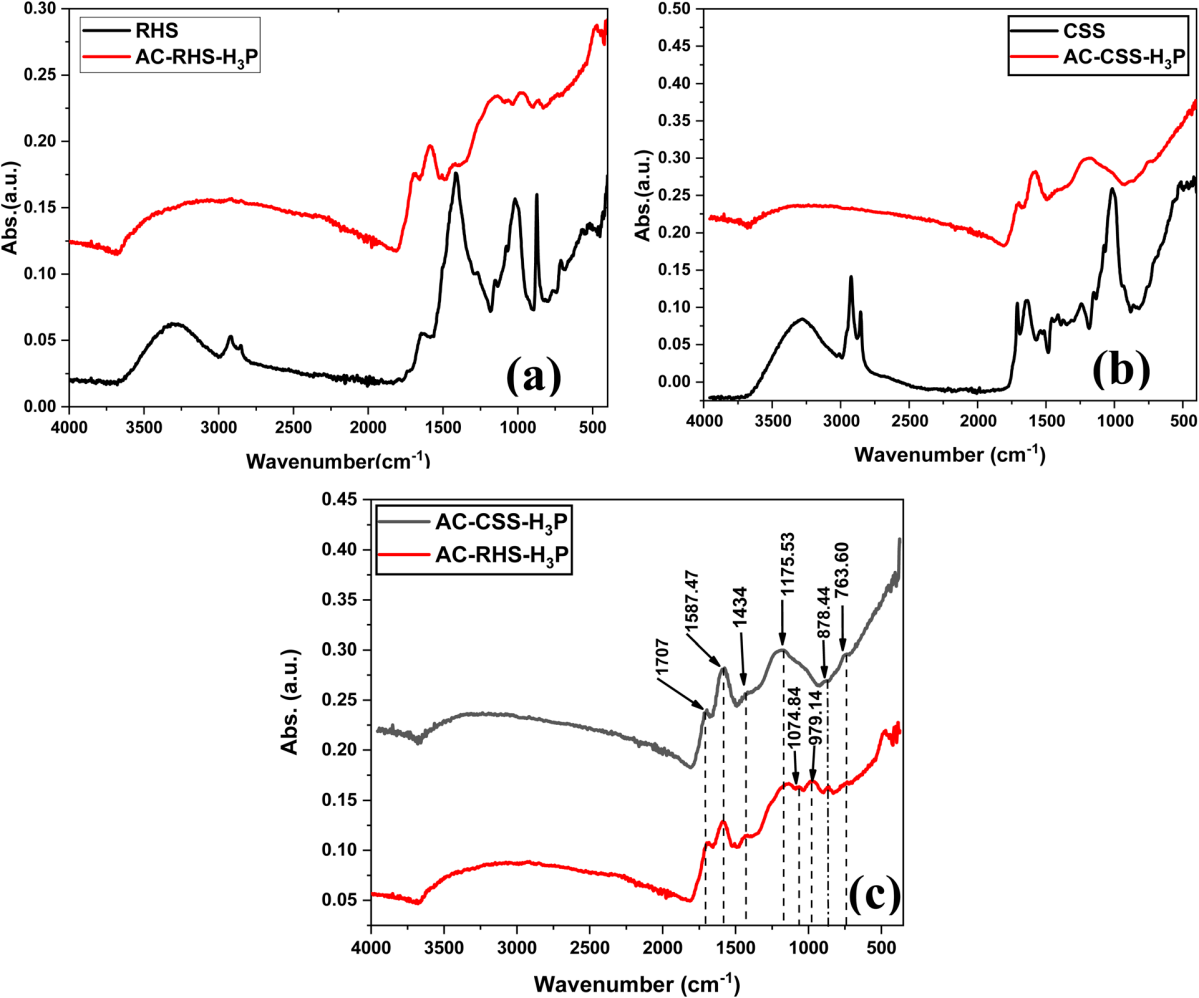
FT-IR comparison spectra of (a) RHS and AC-RHS-H_3_P, (b) CSS and AC-CSS-H_3_P, and (c) AC-CSS-H_3_P and AC-RHS-H_3_P.

Fig. 2 presents the FTIR spectra utilized to ascertain the functional groups present on each activated carbon. The spectra indicate that activation induces significant changes in the surface chemistry of the two biomasses, as evidenced by the disappearance of several characteristic bands and the appearance of new ones (Fig. 2a and b). Moreover, both activated carbons display similar functional groups, as reflected by the strong resemblance between their FTIR spectra (Fig. 2c). The bands observed between 900 and 500 cm^−1^ are ascribed to the out-of-plane deformation vibrations of aromatic C–H bonds on the surface of the activated carbons. The FTIR spectra of the materials also exhibit bands at around 1707 cm^−1^, characteristic of C=O bonds. These bands indicate the presence of carboxylic, ketone, aldehyde, and lactone groups on their surface. Furthermore, the absorption bands at approximately 1175.53 cm^−1^ and 1074.84 cm^−1^ correspond to C–O stretching vibrations, while the bands around 1587.47 cm^−1^ are associated with C=C stretching in aromatic rings. These findings confirm the successful functionalization of the prepared activated carbons and suggest their strong potential to interact with IC molecules through multiple adsorption mechanisms.

#### 3.1.3. SEM results

**Fig. 3:**
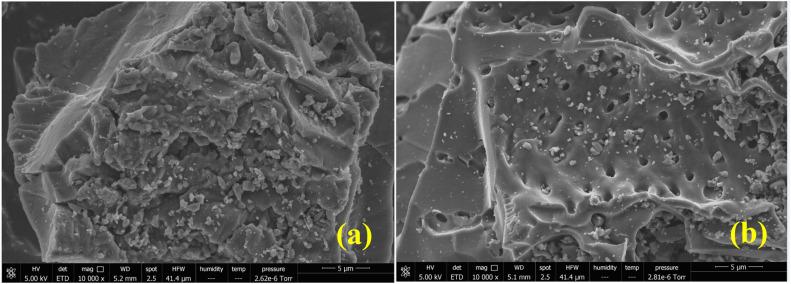
SEM images of the (a) AC-CSS-H_3_P and (b) AC-RHS-H_3_P samples.

The microstructures of the prepared activated carbons, as observed by SEM, are presented in Fig. 3. Both AC-CSS-H_3_P and AC-RHS-H_3_P exhibit heterogeneous surface morphologies with the presence of pores of various sizes. This heterogeneity in the pore structure may facilitate the adsorption of IC from the aqueous solution by providing a broad range of adsorption sites and diffusion pathways. In particular, AC-RHS-H_3_P appears to possess a more developed porosity than AC-CSS-H_3_P, as evidenced by its rougher surface and the predominance of numerous large pores distributed across the carbon matrix. This more extensive pore network is likely to enhance the adsorption capacity of AC-RHS-H_3_P toward IC by increasing the available surface area and pore volume accessible to dye molecules.

#### 3.1.4. Nitrogen adsorption–desorption isotherm analysis

**Fig. 4:**
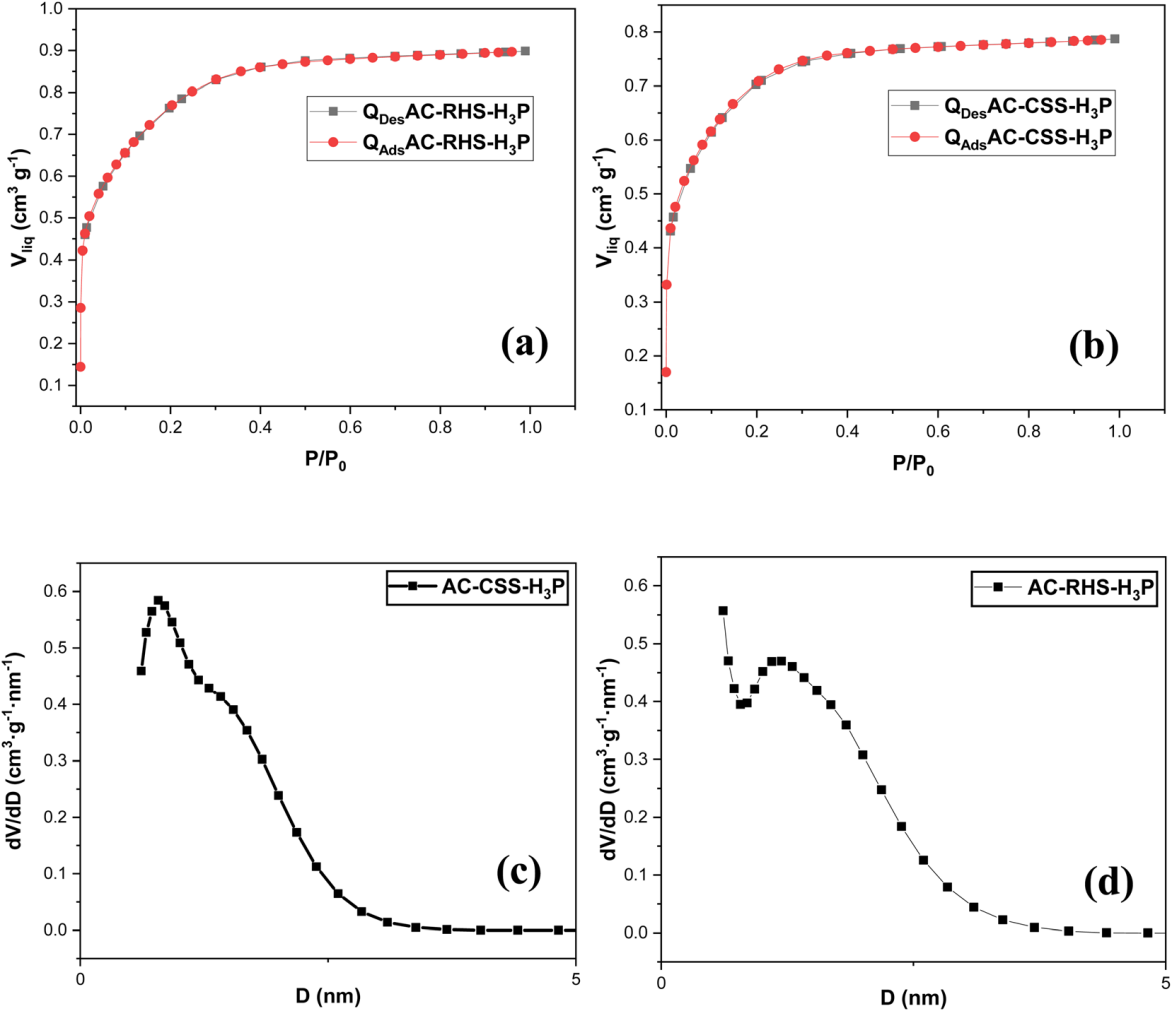
N_2_ adsorption–desorption isotherms (a and b) and QSDFT pore size distribution plots (c and d) of AC-CSS-H_3_P and AC-RHS-H_3_P.

The textural characteristics of the activated carbons were assessed by N_2_ adsorption–desorption analysis. Fig. 4 displays the isotherms and pore size distribution curves. Both materials exhibit type I(b) isotherms (Fig. 4a and b), characteristic of adsorbents containing predominantly large micropores and narrow mesopores.^69^ These observations are supported by the high concentration of pore sizes, obtained by the QSDFT method, in the range from 0.45 to 2 nm and a low concentration in the range from 2 to 4 nm (Fig. 4c and d). Furthermore, according to the isotherms, it appears that the volume of N_2_ adsorbed by AC-RHS-H_3_P is higher, which could reflect more developed surface and volume properties for this sample. This hypothesis is confirmed by the data shown in Table 4. Indeed, the specific surface area values, obtained using the BET model, are estimated to be 1612 and 1696 m^2^ g^−1^ for AC-CSS-H_3_P and AC-RHS-H_3_P, respectively. A similar trend is observed for the specific surface area values obtained by DFT calculations, which are estimated at 1326 and 1504 m^2^ g^−1^, respectively. It is interesting to note that the experimental specific surface areas of the two coals are relatively similar, which may be due to the fact that the volatile matter contents of the two biomass samples are also similar (68.07% and 63.53%). In addition, AC-RHS-H_3_P has a more developed pore volume, with a total volume, *V*_0.95_ (N_2_), of 0.90 cm^3^ g^−1^, compared to 0.78 cm^3^ g^−1^ for AC-CSS-H_3_P, which is consistent with the observations made from the SEM images of the samples. A similar trend is observed with the volumes obtained by DFT calculations. Furthermore, the data presented in Table 4 reveal the microporous nature of the samples. Indeed, the microporous volumes, *W*_0_ (N_2_), cover 75.64% and 66.66% of the total pore volume for the AC-CSS-H_3_P and AC-RHS-H_3_P samples, respectively. In addition, the analyses estimate that the average pore sizes are 1.43 nm and 1.34 nm, respectively. These results correlate with relatively low mesopore volume values. The more developed surface and volume properties of AC-RHS-H_3_P may afford it a greater predisposition to retain IC molecules from the aqueous solution.

**Table 4:** Surface characteristics of AC-CSS-H_3_P and AC-RHS-H_3_P

Sample	pH_PZC_	*S*_BET_ (m^2^ g^−1^)	*S*_DFT_ (m^2^ g^−1^)	*W*_0_ (N_2_) (cm^3^ g^−1^)	*L*_0_ (N_2_) (nm)	*V*_DFT_ (cm^3^ g^−1^)	*V*_0.95_ (N_2_) (cm^3^ g^−1^)	*V*_meso_ (N_2_) (cm^3^ g^−1^)
AC-CSS-H_3_P	5.49	1612	1326	0.59	1.43	0.72	0.78	0.20
AC-RHS-H_3_P	5.43	1696	1504	0.60	1.34	0.82	0.90	0.30

The pH at the point of zero charge (pH_PZC_) of the activated carbons is determined to be 5.49 and 5.43 for AC-CSS-H_3_P and AC-RHS-H_3_P, respectively. These pH_PZC_ values, being below 7, indicate a predominance of acidic functional groups on the surface of both adsorbents. Additionally, the similarity in pH_PZC_ suggests that the two materials may exhibit comparable surface charge behaviors across varying pH conditions. Consequently, at solution pH values below the pH_PZC_, the adsorption of the anionic IC dye onto these activated carbons is expected to be favorable due to the positively charged surface sites.

### 3.2. Analysis of adsorption parameters

#### 3.2.1. Effect of contact time

**Fig. 5:**
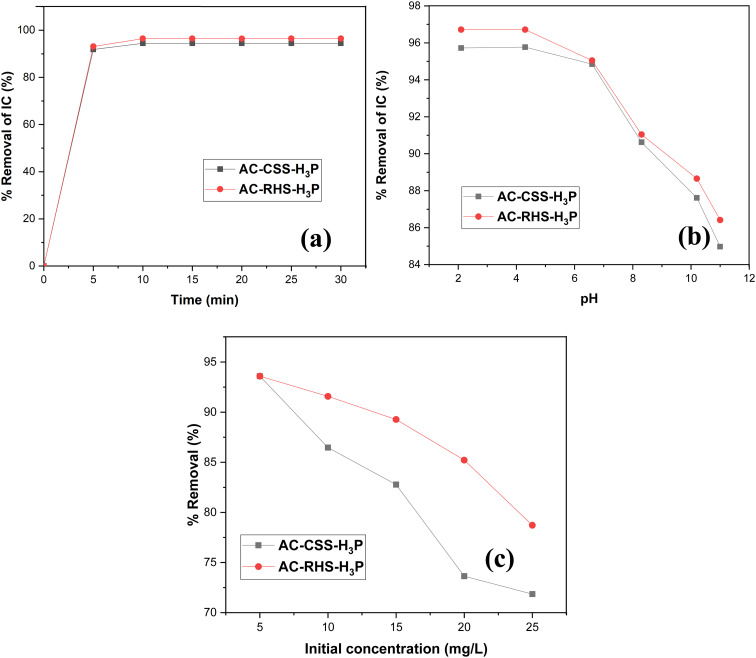
Analysis of the adsorption parameters: (a) effect of the contact time (adsorbent dose = 10 mg; initial IC concentration = 10 mg L^−1^, and *V* = 100 mL), (b) effect of pH (adsorbent dose = 10 mg; initial IC concentration = 10 mg L^−1^; contact time = 10 min; and pH = 2–11), and (c) effect of the initial IC concentration (pH = 4.0; adsorbent dose = 10 mg; contact time = 10 min; and initial concentration = 5–25 mg L^−1^) on the percentage removal of the IC dye using AC-CSS-H_3_P and AC-RHS-H_3_P.

To examine the influence of the contact duration, studies were performed across a time span of 0–30 min utilizing an adsorbent dosage of 10 mg and an initial concentration of IC dye of 10 mg L^−1^. As shown in Fig. 5a, the adsorption process exhibits three distinct stages. The initial stage is marked by a rapid uptake of IC, due to the plentiful active adsorption sites present.^70,71^ During this stage, a linear increase in IC removal is observed, lasting approximately 5 min. The second stage, occurring between 5 and 10 min, shows a slight reduction in the adsorption rate, which may be due to the progressive occupation of active sites and the decreasing concentration of IC in the solution. The third stage begins after 10 min, where an equilibrium state is achieved, likely due to the saturation of the remaining adsorption sites. At equilibrium, the IC removal efficiencies reach 94% and 96% for AC-CSS-H_3_P and AC-RHS-H_3_P, respectively. Therefore, 10 min was selected as the optimal equilibrium contact time for the subsequent experiments.

#### 3.2.2. Effect of pH

The experiments to determine the optimal pH for IC dye removal were conducted for a fixed contact time of 10 min at an adsorbent dose of 10 mg while varying the pH from 2 to 11. For each experiment, the pH of the dye solution was adjusted to the desired value before adding the adsorbent and initiating stirring. As shown in Fig. 5b, for both adsorbents, the maximum removal efficiency is achieved in the pH range of 2–4, followed by a slight decrease in removal efficiency at pH values above 5. A similar trend, with the optimum IC removal near pH 2, has been reported by other authors.^71,72^ The high removal efficiency in the acidic pH range (2–4) can be explained by the pH_PZC_ values of the materials (5.43 and 5.49 for AC-CSS-H_3_P and AC-RHS-H_3_P, respectively). The surface charge of the adsorbents becomes positive below the pH_PZC_, promoting attraction of the anionic IC dye species, whereas at pH values above the pH_PZC_, the surface becomes negatively charged. Consequently, under basic conditions, electrostatic repulsion occurs, limiting IC adsorption and resulting in a reduced removal efficiency.^23^ Furthermore, the high removal efficiency observed under acidic conditions can be attributed to the favorable interactions between the adsorbents and IC molecules, particularly through hydrogen bonding between the acidic surface functional groups of AC-CSS-H_3_P and AC-RHS-H_3_P and the nitrogen- and oxygen-containing functional groups of the IC molecule.^11,64,73^ At pH > 6.8, the excess hydroxide ions in the solution impart negative charges to the surface of both activated carbons, leading to electrostatic repulsion with the anionic IC species.^11^ As a result, the negatively charged surface sites do not favor IC adsorption due to this repulsive interaction.^3^ In addition, the competition between IC anions and hydroxide ions for the same adsorption sites, coupled with the predominance of weaker van der Waals forces and physical interactions, further contributes to the decrease in removal efficiency under alkaline conditions. Similar observations have been reported by other authors for IC adsorption^5,11,71,73^ and for Congo red adsorption on cashew nut shells.^3,74^

#### 3.2.3. Effect of initial concentration

To evaluate the effect of the initial IC concentration on dye removal, the previously established optimal conditions were applied, namely, an adsorbent dose of 10 mg, a pH of 4.0, and a contact time of 10 min, while the initial dye concentration was varied from 5 to 25 mg L^−1^. As illustrated in Fig. 5c, the percentage removal of IC decreases with increasing initial dye concentration for both activated carbons, indicating that this parameter significantly influences the adsorption process. Specifically, the removal efficiency decreases from 93.58% to 71.85% for AC-CSS-H_3_P and from 93.58% to 78.71% for AC-RHS-H_3_P as the initial concentration increases from 5 to 25 mg L^−1^. This decline in percentage removal is due to the restricted availability of active sites on the adsorbent surface; as the dye concentration rises, the available sites become saturated, resulting in a lower removal efficiency. At lower concentrations, a greater proportion of dye molecules can access and occupy active sites, leading to higher removal. Similar behavior has been reported by Senthil *et al.*^3^ for the adsorption of Congo red dye onto cashew nut shells.

### 3.3. Analysis of adsorption isotherms

**Fig. 6:**
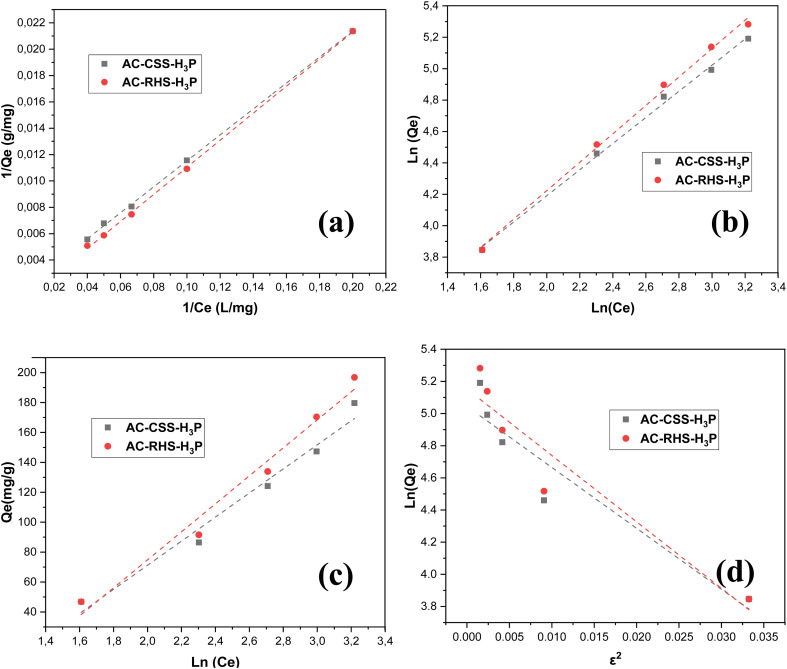
Isotherm modeling plots of IC removal using AC-CSS-H_3_P and AC-RHS-H_3_P (*C*_0_ = 10 mg L^−1^; contact time = 10 min; mass weight = 10 mg, and pH = 4.0): (a) Langmuir model, (b) Freundlich model, (c) Temkin model, and (d) D–R model.

The adsorption isotherms were evaluated by fitting the experimental data to the Langmuir, Freundlich, Temkin, and D–R models. Accordingly, the linear plots of 1/*Q*_e_*versus* 1/*C*_e_, ln(*Q*_e_) *versus* ln(*C*_e_), *Q*_e_*versus* ln(*C*_e_), and ln(*Q*_e_) *versus ε*^2^ for IC adsorption onto AC-CSS-H_3_P and AC-RHS-H_3_P are presented in Fig. 6. These linear transformations with respect to the equilibrium concentration were employed to determine the corresponding adsorption parameters (Table 5). The adequacy of the experimental data for each adsorption model was evaluated based on the correlation coefficient (*R*^2^). An inspection of Table 5 indicates that the four isotherm models examined fit the experimental data well. A similar observation was reported in our previous work on the adsorption of IC onto cola nut shells.^21^ However, the best fit is observed for the Langmuir model, which yields the highest *R*^2^ value. This model states that the maximum adsorption is achieved when a saturated monolayer of solute molecules covers the adsorbent surface.^53^ A comparison of the adsorption capacities derived from this model with the data from the literature (Table 6) highlights the remarkable performance of the materials studied. Indeed, the maximum IC retention capacities obtained, 588.24 mg g^−1^ for AC-CSS-H_3_P and 1250.00 mg g^−1^ for AC-RHS-H_3_P, are significantly higher than those reported for other adsorbents. The high adsorption capacity of the adsorbents is attributable to their well-developed porosity and abundant surface functional groups. Moreover, this conclusion is supported by the values of the separation factor, *R*_L_ (0.7547 and 0.8696), which fall within the range of 0–1, indicating the favorable adsorption of IC onto AC-CCS-H_3_P and AC-RHS-H_3_P, respectively. Specifically, comparable maximum adsorption capacities have been reported in previous studies, including those by Kekes *et al.*^75^ on chitosan and chitosan/β-cyclodextrin cross-linked bead particles, Bessaha *et al.*^76^ on calcined Zn/(Al + Fe) layered double hydroxides, Jésica Trujillo-Reyes *et al.*^22^ on Fe–Ni nanostructures, and Gad *et al.*^31^ on apricot-stone-derived activated carbon, as summarized in Table 5. However, the aforementioned studies, with the exception of that of Gad *et al.*,^31^ prepared their materials for IC removal from aqueous solutions using precursors other than lignocellulosic biomass. For the Freundlich model, the obtained 1/*n* values (0.832 and 0.9048) indicate the heterogeneous surface characteristics of AC-CSS-H_3_P and AC-RHS-H_3_P, respectively.^55^ The values of *K*_F_ (0.0799 and 0.0895 mg g^−1^), which are related to the bonding energy, suggest that a chemisorption process occurs, further supported by the high *R*^2^ values (>0.99) close to unity. For the Temkin and D–R models, IC adsorption onto both activated carbons shows a moderate fit, with *R*^2^ values ranging from 0.907 to 0.9775. This finding further supports the idea of adsorption occurring at energetically heterogeneous sites.

**Table 5:** Parameters of the adsorption isotherms

Isotherms	Parameters	AC-CSS-H_3_P	AC-RHS-H_3_P
Langmuir	*Q*_max_ (mg g^−1^)	588.24	1250.00
	*K*_L_ (L mg^−1^)	0.0162	0.0075
	*R*_L_	0.8606	0.9302
	*R* ^2^	0.999	0.999
Freundlich	1/*n*	0.832	0.9048
	*K*_F_ (mg g^−1^)	0.0799	0.0895
	*R* ^2^	0.997	0.997
Temkin	*B*_T_ (J mol^−1^)	80.547	93.631
	*K*_T_ (L g^−1^)	3.0527	3.3234
	*R* ^2^	0.975	0.978
D–R	*B*_D_ (mol^2^ kJ^−2^)	41.322	37.905
	*Q*_D_ (mol^2^ kJ^−2^)	172.8977	155.1202
	*E* (J mol^−1^)	0.1100	0.1149
	*R* ^2^	0.907	0.903

**Table 6:** Comparison of the maximum adsorption capacities of dyes onto other materials

Adsorbents	Maximum adsorption capacities (mg g^−1^)	Surface area (m^2^ g^−1^)	Dominant adsorption mechanism	References
Carbon nanotubes	136.00	145.90	Chemisorption	10
CuAl-LDH/SWCNT nanocomposite	297.12	—	—	11
Chitosan/activated carbon composite	208.33	256.82	Physisorption	12
Cobalt hydroxide nanoparticles	163	—	—	13
Montmorillonite	40.00	73.916	—	15
Cola nut shells	10.22	—	Chemisorption	21
Fe–Ni nanostructures	977.18	134.11	Chemisorption	22
ACZ/AgNP	411.68	—	Chemisorption	23
ACZ	121.00	—	Chemisorption	23
Mg/Fe layered double hydroxide nanoparticles	62.80	85.5	Chemisorption	24
Chitosan hydrogel/Hyper-crosslinked polymer particles	118.00	—	—	25
Apricot stone activated carbon	552.55	98	Chemisorption	31
Maize cob carbon	118.48	809.8	Physisorption	32
Commercial activated carbon	298.34	1250.320	Chemisorption	33
Pomegranate peel (APP)	158.73	51.0674	Physisorption	71
Chitosan	500.00	—	Chemisorption	75
Chitosan/β-cyclodextrin crosslinked bead particles	1000.00	—	Chemisorption	75
Calcined (Zn/Al + Fe) layered double hydroxide	617.3	53.29	—	76
Nanofiber membranes	266.77	140.1	Chemisorption	77
*Acacia nilotica* (babool) sawdust activated carbon	37.91	—	—	78
Magnesium oxide nanoparticles	559.2	127.442	Chemisorption	79
PKSAC	11.03	574.500	Chemisorption	80
PKSAC/BVA	12.64	331.899	Chemisorption	80
MgOBi_2_	126	12.2	Chemisorption	81
AC-CSS-H_3_P	588.24	1612	Chemisorption	This work
AC-RHS-H_3_P	1250.00	1696	Chemisorption	This work

### 3.4. Analysis of adsorption kinetics

**Fig. 7:**
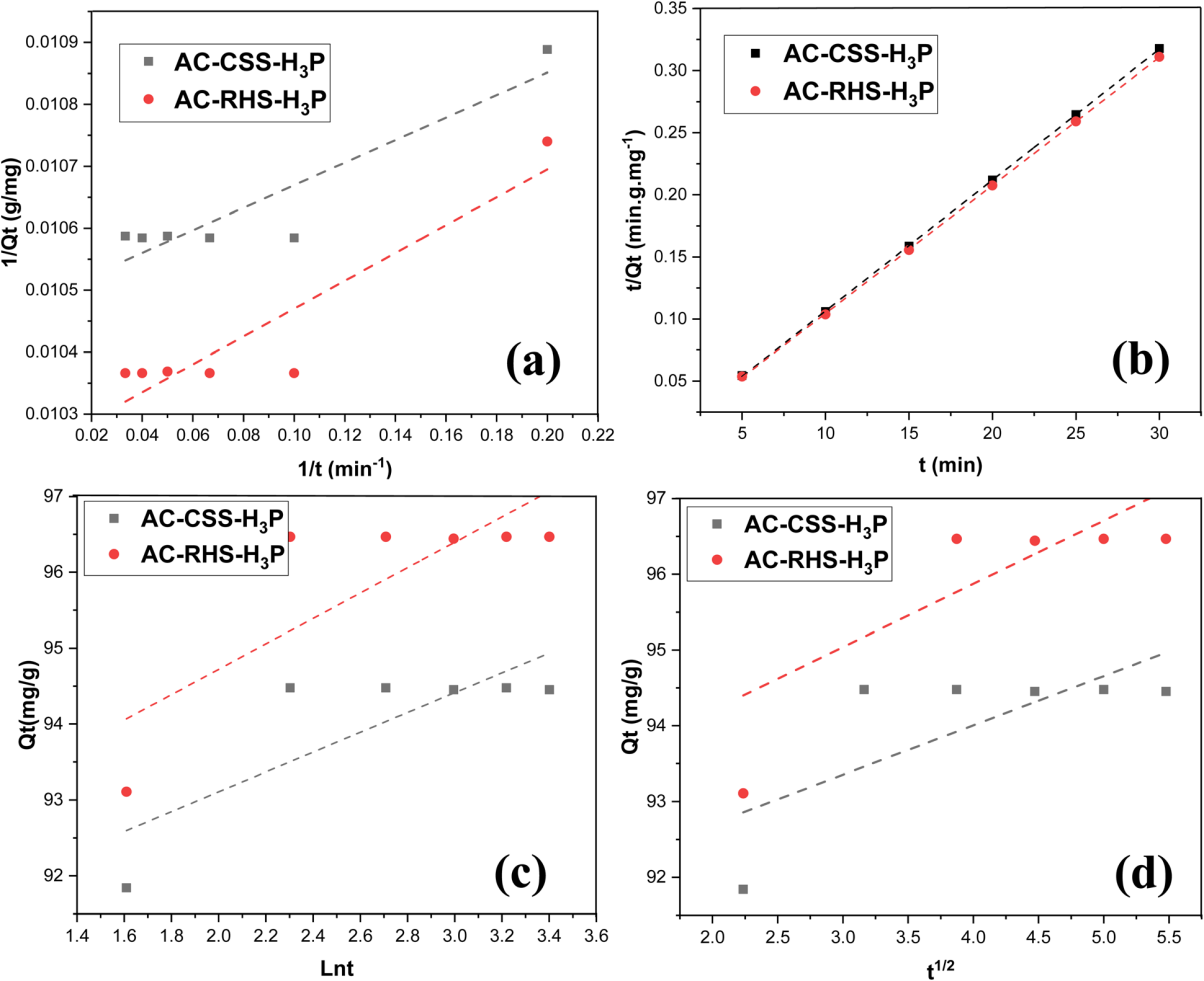
Plots of the kinetics models for IC removal using AC-CSS-H_3_P and AC-RHS-H_3_P: (a) pseudo-first order, (b) pseudo-second order, (c) Elovich, and (d) intraparticle diffusion models.

**Fig. 8:**
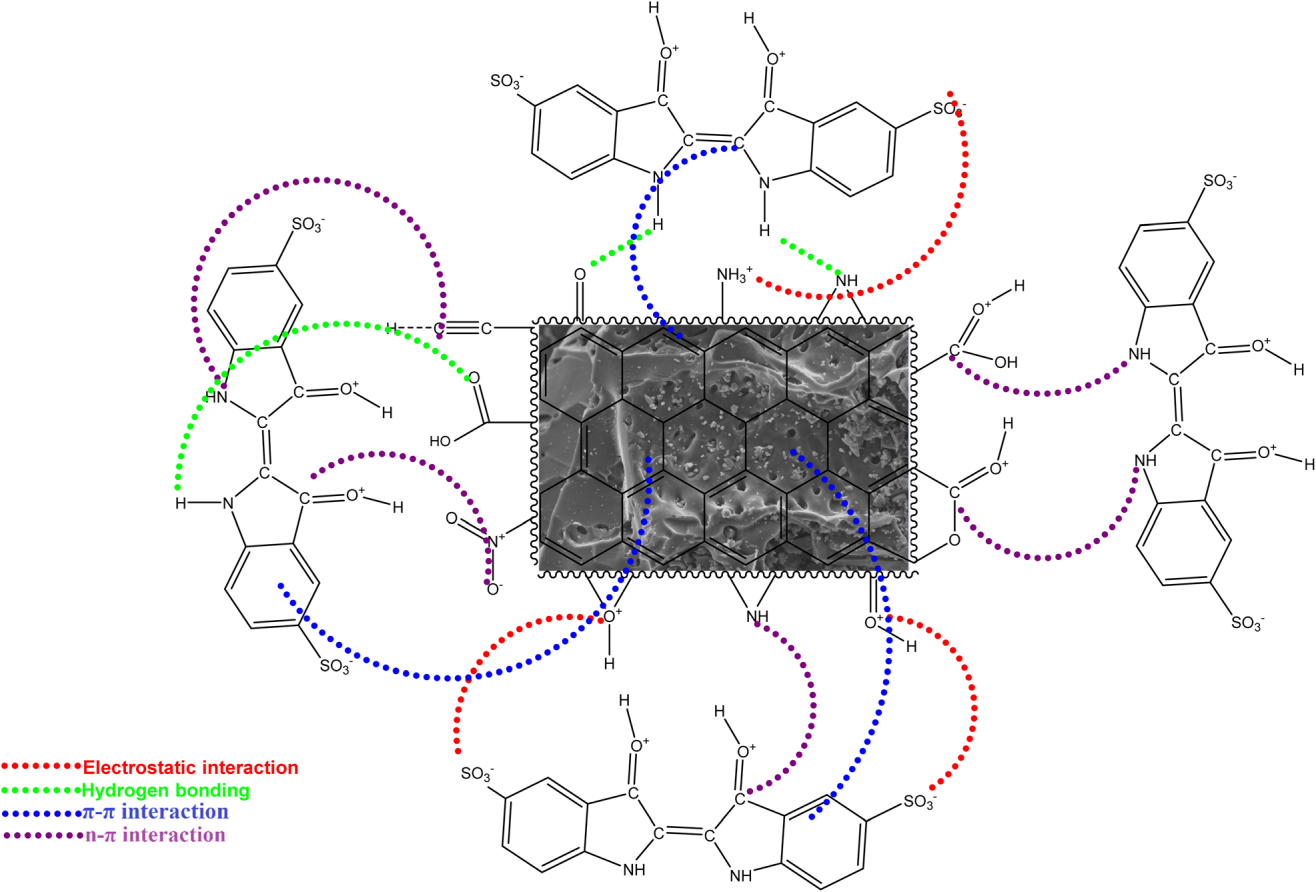
Probable mechanism of the IC dye adsorption on AC-CSS-H_3_P and AC-RHS-H_3_P.

The experimental data were fitted to the pseudo-first-order, pseudo-second-order, Elovich, and intraparticle diffusion kinetic models. The corresponding linear plots as a function of time are presented in Fig. 7, and the kinetic parameters are summarized in Table 7. The suitability of each kinetic model was evaluated based on the correlation coefficient (*R*^2^), which should ideally be close to unity. The plot of *t*/*Q*_*t*_*versus t* produced a straight line with an *R*^2^ value of 0.999 for both adsorbents, indicating the excellent fit of the pseudo-second-order model to the experimental data. This finding clearly demonstrates that chemisorption is the rate-limiting step governing IC adsorption onto AC-CSS-H_3_P and AC-RHS-H_3_P. In this context, the adsorption process relies primarily on π–π and n–π interactions between the functional groups on the surface of the adsorbents and those of the dye. However, based on the variation in the adsorbed amount at different pH levels, it is clear that other interactions, particularly electrostatic interactions, contribute to the adsorption process. Furthermore, hydrogen bonds are likely to form between the oxygen- and nitrogen-containing functional groups present in the molecular structure of the IC and the acidic groups present on the adsorbents, and *vice versa*. Accordingly, based on the *R*^2^ values, the kinetic behavior of IC adsorption onto AC-CSS-H_3_P and AC-RHS-H_3_P follows the order: intraparticle diffusion < Elovich < pseudo-first-order < pseudo-second-order. Fig. 8 illustrates the interactions involved in the adsorption of IC onto the activated carbons.

**Table 7:** Parameters of the adsorption kinetics

Kinetic model	Parameters	AC-CSS-H_3_P	AC-RHS-H_3_P
PFO	*Q*_1_ (mg g^−1^)	9.5238	98.0392
	*K*_1_ (min^−1^)	0.0171	0.2157
	*R* ^2^	0.851	0.855
PSO	*Q*_2_ (mg g^−1^)	95.2381	97.0874
	*K*_2_ (g mg^−1^ min^−1^)	0.0005	0.3536
	*R* ^2^	0.999	0.999
Elovich	*Β* (g mg^−1^)	0.7658	0.5974
	*α* (mg g^−1^ min^−1^)	1.6362 × 10^30^	8.4993 × 10^23^
	*R* ^2^	0.651	0.657
Intraparticle	*k*_P_ (mg (g min^1/2^)^−1^)	0.6506	0.8353
	*C* (mg g^−1^)	91.4040	92.5320
	*R* ^2^	0.532	0.538

### 3.5. Regeneration test of AC-CSS-H_3_P and AC-RHS-H_3_P

**Fig. 9:**
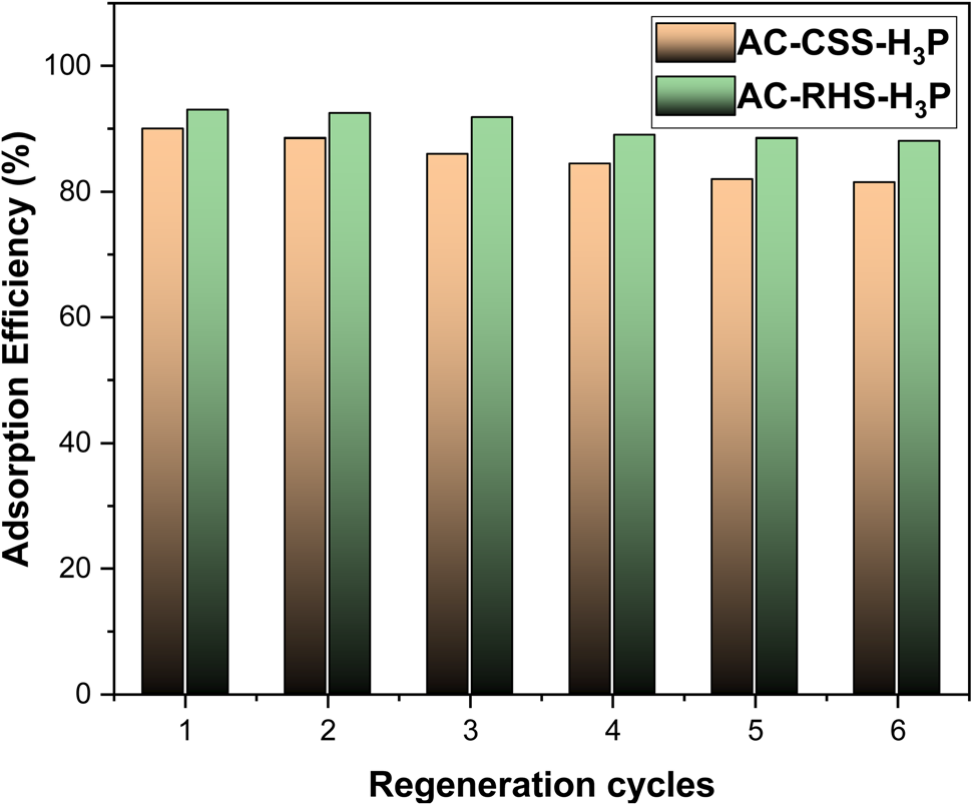
Regeneration cycles of AC-CSS-H_3_P and AC-RHS-H_3_P.

In accordance with the observation of reduced IC adsorption under basic conditions, desorption tests were conducted using a low-concentration sodium hydroxide solution to release the adsorbed IC. Each IC-loaded adsorbent was stirred in 100 mL of a 4 mmol L^−1^ NaOH solution for 60 min. Subsequently, the chemically regenerated adsorbent was subjected to IC adsorption again to evaluate its reusability under the same optimized conditions (initial concentration = 10 mg L^−1^, adsorbent dose = 10 mg, pH = 4, and contact time = 15 min). As illustrated in Fig. 9, the IC removal efficiency remained nearly constant for the first three cycles and then stabilized over the final three cycles for AC-RHS-H_3_P. In contrast, AC-CSS-H_3_P exhibited a slight decrease in the IC removal efficiency during the first four cycles, after which performance plateaued, indicating the partial exhaustion of adsorption sites and effective regeneration. Both adsorbents maintained high adsorption efficiency, with no significant loss in performance observed after six regeneration cycles. From an economic perspective and based on the regeneration test results, these findings confirm that AC-CSS-H_3_P and AC-RHS-H_3_P are sustainable and low-cost adsorbents suitable for the treatment of organic dye-contaminated effluents.

## 4. Conclusion

This study demonstrated the suitability of CSS and RHS as effective precursors for the preparation of activated carbons and their strong adsorption capability for IC removal from aqueous solutions. The presence of abundant surface functional groups and a well-developed mesoporous structure played a key role in the high IC removal efficiencies achieved. IC adsorption was found to be unfavorable under basic conditions, whereas acidic pH significantly enhanced adsorption performance. Although all four isotherm models adequately described the equilibrium behavior, the pseudo-second-order kinetic model provided the best fit to the experimental data, indicating that chemisorption was the rate-limiting mechanism governing IC adsorption. The chemisorption process was attributed to the formation of hydrogen bonds between the oxygen and nitrogen atoms in the IC molecule and the acidic functional groups on the activated carbon surfaces. Overall, the CSS- and RHS-derived activated carbons showed excellent reusability and adsorption efficiency, confirming their potential as low-cost, sustainable adsorbents for the efficient removal of organic dyes from aqueous media.

## Author contributions

Daouda Kouotou: writing – original draft, visualization, validation, resources, methodology, investigation, formal analysis, data curation, and conceptualization. Frank Dorinel Solefack Feudjio: writing – original draft, visualization, methodology, investigation, data curation, and formal analysis. Abdelhakim Elmouwahidi, Murat Yilmaz and Julius Nsami Ndi: visualization, validation, writing – review and editing, methodology, data curation, and formal analysis. Agustin F. Perez-Cadenas and Francisco Carrasco-Marin: validation, supervision, writing – review and editing, resources, and project administration.

## Conflicts of interest

The authors declare no conflicts of interest.

## Data Availability

Data are available on request.
